# Air Corridors: Concept, Design, Simulation, and Rules of Engagement

**DOI:** 10.3390/s21227536

**Published:** 2021-11-12

**Authors:** Sabrina Islam Muna, Srijita Mukherjee, Kamesh Namuduri, Marc Compere, Mustafa Ilhan Akbas, Péter Molnár, Ravichandran Subramanian

**Affiliations:** 1 Electrical Engineering Department, University of North Texas, Denton, TX 76203, USA; sabrinaislammuna@my.unt.edu; 2 Harbor Branch Oceanographic Institute, Florida Atlantic University, Boca Raton, FL 33431, USA; srijitamukherjee@my.unt.edu; 3 Department of Mechanical Engineering, Embry-Riddle Aeronautical University, Daytona Beach, FL 32114, USA; comperem@erau.edu; 4 Department of Electrical Engineering and Computer Science, Embry-Riddle Aeronautical University, Daytona Beach, FL 32114, USA; akbasm@erau.edu; 5 Amazon Web Services, Atlanta, GA 98170, USA; molnarp@amazon.com; 6 Robinson College of Business, Georgia State University, Atlanta, GA 30302, USA; 7 Hermes Autonomous Air Mobility Solutions, Dallas-Fort Worth, TX 75225, USA; ravisub@gmail.com

**Keywords:** air corridors, unmanned air vehicle, vehicle-to-vehicle communications, geofence, capacity, collision-avoidance

## Abstract

Air corridors are an integral part of the advanced air mobility infrastructure. They are the virtual highways in the sky for the transportation of people and cargo in a controlled airspace at an altitude of around 1000 ft. to 2000 ft. above ground level. These corridors will be utilized by (unmanned) air taxis, which will be deployed in rural and metropolitan regions to carry passengers and freight, as well as air ambulances, which will be deployed to offer first responder services such as 911 emergencies. This paper presents fundamental insights into the design of air corridors with high operational efficiency as well as zero collisions. It begins with the definitions of air cube, skylane or track, intersection, vertiport, gate, and air corridor. Then a multi-layered air corridor model is proposed. Traffic at intersections is analyzed in detail with examples of vehicles turning in different directions. The concept of capacity of an air corridor is introduced along with the nature of distribution of locations of vehicles in the air corridor and collision probability inside the corridor are discussed. Finally, results of traffic flow simulations are presented.

## 1. Introduction

Air corridors are three-dimensional (3D) volumes of airspace reserved for Unmanned Aircraft Systems (In this paper, the terms UAS and Unmanned Aerial Vehicle (UAV) are used interchangeably for convenience. However, by definition, a UAS includes a UAV, its ground control station, and a human operator.) (UASs) for Advanced Air Mobility (AAM) traffic [1,2]. Air corridor design specifications are specific to each country and are defined by its respective federal aviation authority. In the United States, the Federal Aviation Administration (FAA) defines air corridors in class B, C, or D airspace [3]. The definition of an air corridor is flexible in nature. FAA has the right to open or close an air corridor. The FAA also defines the expected performance requirements of a UAS flying in an air corridor. The design of air corridors, traffic rules in air corridors, safety requirements, and performance specifications are still evolving. Airspace design concepts, such as geofences [4], are currently being considered by various research groups.

### 1.1. Major Contributions

This paper takes a first attempt to the design of air corridors with high efficiency as well as zero collisions. It begins with the definitions of an air cube, skylane or track, intersection, vertiport, gate, and air corridor. Then a multi-layered air corridor model is proposed. Traffic at intersections is analyzed in detail, with examples of vehicles turning in different directions. The concept of capacity of the air corridor, location distribution of vehicles and collision probability inside the corridor are discussed. Finally, results of traffic flow simulations are presented.

### 1.2. Organization

This paper is organized as follows: Section 2 gives a summary of recent developments in this topic. Section 3 provides definitions and notations used in this paper. This section begins with the existing definition of geofences [4], and derives several new definitions including an air cube, skylane, intersection, vertiport, gate, and air corridor. Section 4 presents the design and traffic management method of multi-layered air corridors. The strategies for handling traffic at intersections are discussed with examples at Section 5. Capacity in terms of the number of UAVs as well as estimated travel time is presented at Section 6. Section 7 discusses the mobility model and the probability density function of the vehicle locations in a skyline. Section 8 derives the probability of collision in a skylane. Section 9 displays the results of simulations of various traffic volumes and UAV speeds. Section 10 concludes the paper with summary and final thoughts.

## 2. Literature Review

There are numerous applications for UAVs in military and civilian fields. Sample applications include: (1) providing communication services by serving as base stations (BSs) [5,6], (2) monitoring air pollution or toxic gas leakage [7,8], (3) transporting cargo [9], (4) disaster prediction, assessment, and response [5], and (5) providing cost-effective wireless connectivity for devices without infrastructure coverage [10].

In the near future, (unmanned) air taxis are expected to be deployed in rural and urban areas to transport people and cargo from one place to another. Air ambulances are expected to be deployed to provide first responder services. Unlike commercial flights, UAVs are highly flexible with uncertain movement patterns, which makes UAV traffic analysis more challenging. UAS Traffic Management (UTM) systems are concerned with more than just regulations; they are also concerned with safety and efficiency. Further, UAV traffic needs to be restricted from flying above private properties, parks, highways, and other heavily populated areas. As a result, pre-defined flight plans, and deterministic flight trajectories are needed because of the limitation of airspace. In [11] the authors describe how less structured airspaces with free flight movement allow for greater capacity and route efficiency. They also point out that free flights require greater technological capabilities and compromise safety. On the other hand, more restrictive structures, such as skylanes, enable the operations of less-equipped aircraft at the expense of slightly increased delays. Designated sections of controlled airspace, which are known as air corridors, not only improve safety and assure compliance but also enable higher tempo Urban Air Mobility (UAM) operations while trying to minimize the impact on other air traffic. Corridors could have multiple routes with different performance requirements, which provide predictability and structure, increase throughput and minimize bottlenecks. Due to the well-defined structure of an air corridor, the overheads such as communication, handovers, traffic management for UAVs flying in an air corridor are minimal as compared to those that are flying outside the air corridor [2].

Aircraft systems operating in air corridors follow specific procedures defined for these corridors. Some specific characteristics, such as routes, are static but used in a flexible fashion. FAA controls which routes are open or closed for traffic at any given time. Some design considerations for the route selection include airspace class, noise levels, departure/approach flows, final approach paths, etc. Routes are charted and made available to other airspace users and are periodically redefined as operational demand changes [12].

## 3. Definitions and Notations

Airspace is organized in terms of its building blocks—air cubes, skylanes, geofences, intersections and air corridors. The notation followed to represent these building blocks is shown on Table 1. The mathematical definitions of these building blocks will be discussed in this section. UASs also require vertiports or vertical airports for takeoff and landing operations. As a result, typical UAVs that are allowed to fly in air corridors are electric vertical takeoff and landing vehicles.

**Definition** **1** (Geofence, [4])**.** *In the context of UAS, the term geofencing is used to describe virtual three dimensional “boundaries” each UAS flies within or avoids as a no-fly zone (NFZ).*

(1) $$g=\left\{n,v\left[\;\right],z_{f},z_{c},m,h\left[\;\right],ids\left[\;\right]\right\}$$

*A geofence (g) is a volume defined by a minimum floor altitude $z_{f}$, maximum ceiling altitude $z_{c}$, and a list of n horizontal vertices $v=[\left(x_{1},y_{1}\right),\left(x_{2},y_{2}\right),....,\left(x_{n},y_{n}\right)]$ where n≥3. The volume is defined relative to the set of home locations, $h_{i}=\left(\phi_{i},\lambda_{i},z_{i},t_{i}\right)$ where h[] is a list of length m≥2. The pair $\left(\phi_{i},\lambda_{i}\right)$ represent the respective latitude and longitude of the home location. The altitude of the home location above mean sea level (MSL) is represented by $z_{i}$ and the activation time for home location i for 1≤i<m is represented by $t_{i}$. The variable $t_{m}$ is the deactivation time for geofence g. Permission to enter and operate within geofence g is indicated by ids[], which contains a list of the unique identification numbers for all permitted UAV. Geofence boundaries are defined in meters relative to a home location as a vertical floor $z_{f}$ and ceiling $z_{c}$ and a list of vertices $v=v_{1},...,v_{n}$ for each $v_{i}=\left(x_{i},y_{i}\right)$, i=1,...,n where n is the number of vertices. The vertices define a closed simple polygon parallel to the horizontal plane. There is no convexity requirement on the polygon. The polygon is extruded to the vertical limits of the geofence to construct the geofence volume. The horizontal vertices and vertical limits are constant relative to a sequence of one or more home locations defined in set h[].*

*There are two types of geofences - static and dynamic. A static geofence is always active and represents unchanging boundaries such as international borders, property lines, buildings, utility poles and lines, and 24-hour airport final approach and initial departure corridors. On the other hand, a dynamic geofence is not necessarily always active, and the home location is not necessarily constant.*

**Definition** **2** (Air Cube)**.** *An air cube (c) is a building block for a skylane in 3D airspace. An air cube is a static geofence in its most simplified form. All air cubes are similar in size. An air cube is exclusively reserved space for a UAV in transit at any given time. According to Near Mid-Air-Collision (NMAC) avoidance rules, the standard safe distance between two manned aircraft is 500 ft. or 152.4 m [13]. In our model, the side length (s) of each air cube is set to 200 m.*

(2) $$c\left[cid\right]=\left\{cid,center,s,direction\right\}$$

*Each air cube has a unique identifier cid which helps UAVs to understand the cube occupancy information while moving. $center=\left\{\phi ,\lambda ,z_{c}\right\},$ indicates the center point of the air cube. ϕ, λ, $z_{c}$ represent latitude, longitude, and ceiling altitude, respectively. From the the side length(s) and the center of the air cube, the overall air cube volume can be determined. Direction indicates the traffic flow direction inside the cube.*

**Definition** **3** (Skylane or Track)**.** *A skylane (S) is a designated region of airspace for UAV (Unmanned Air Vehicle) in transit. An aircraft must fly within the skylane during its transit. A skylane can be defined as a volume consisting of a number of cubes with the same direction. An entrance gate is used to enter the skylane and one exit gate is used to exit the skylane. Gates are defined in Definition 6.*

(3) $$S\left[sid\right]=\left\{sid,direction,nc,c[cid],gates\right\},$$

*where sid represents the unique identifier of the skylane, direction represents one of the the four directions of travel (East-to-West), (West-to-East), (South-to-North), or (North-to-South). This paper considers three layered air corridors in a typical urban or rural setting, each layer consisting of two skylanes. The top layer contains two one-directional skylanes: South-to-North and North-to-South. The bottom layer contains two one-directional skylanes: East-to-West and West-to-East. The middle-layer is used by UAVs to make turns.*

**Figure 1:**
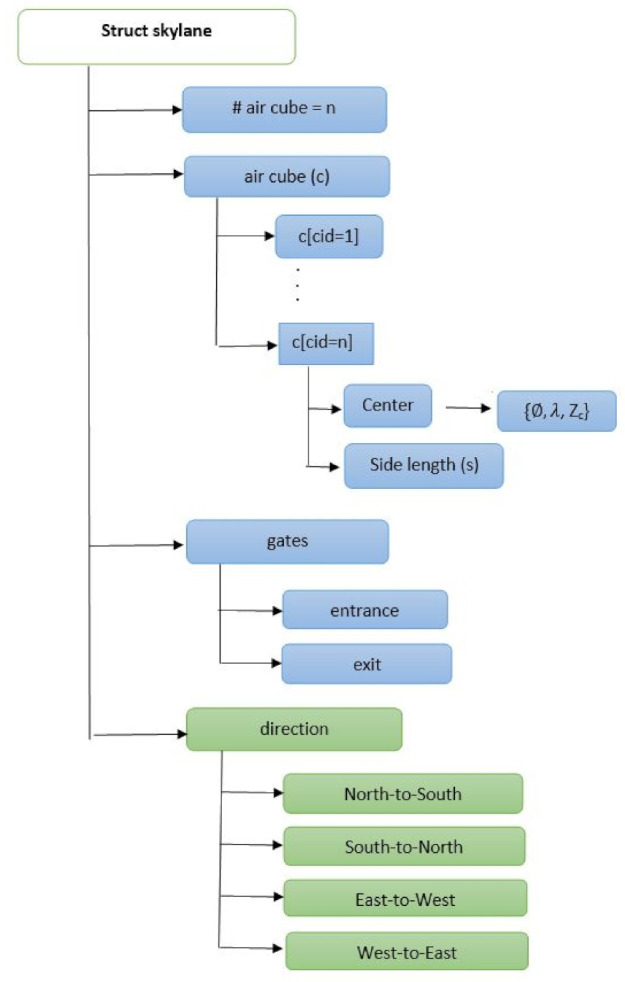
Skylane structure. Blue boxes represent variables that define the data elements of skylane. Green boxes represent variables that define movement directions of vehicles inside the skylane.

*Figure 1 illustrates a data structure of a skylane where blue boxes represent variables that specify skylane data items such as air cube, gate, and so on. Green boxes represent variables that specify vehicle movement directions within the skylane. The length of the skylane, L is equal to nc×s, where s is the side length of an air cube defined in meters and nc is the number of air cubes.*

**Definition** **4** (Intersection)**.** *An intersection is the junction where one skylane crosses another in the horizontal plane. In the skylane, it is the place where vehicles turn or change their direction. In order to avoid collisions, an intersection is designed to include three layers. The middle layer is used for a temporary hovering before a UAV actually makes the intended turn.*

**Definition** **5** (Vertiport)**.** *A vertical airport or vertiport (V) is a place for take-off and landing for UAVs.*

(4) $$V\left[vpid\right]=\left\{vpid,v\left[\;\right],z_{c},ids\left[\;\right]\right\}$$

*Each vertiport has a unique identifier vpid. The volume of a vertiport is defined by its horizontal vertices $v[\left(x_{1},y_{1}\right),\left(x_{2},y_{2}\right),\left(x_{3},y_{3}\right),\left(x_{4},y_{4}\right)]$ and its maximum ceiling altitude $z_{c}$. The array ids[] is the sequence of identification numbers of the UAVs that are permitted to land or take off from the vertiport.*

**Definition** **6** (Gate)**.** *A gate is a connection between a skylane and a vertiport. It regulates the takeoff and landing operations of the UAVs. Vehicles need to go through the gates to enter or exit the skylanes.*

**Definition** **7** (Air corridor)**.** *An air corridor is a 3D volume of airspace reserved for UASs. It is a complete airspace structure that includes all skylanes, intersections, and gates. Cube, skylanes, and air corridor definitions assume end-to-end cube placement for a continuous segment but real GPS signals with uncertainty will require clear rules for unambiguous cube occupancy.*

## 4. Air Corridor Design and Rules of Engagement

In this section, the design of a multi-layered air corridor, traffic coordination, and the rules of engagement in air corridors are described. This section also illustrates through few examples how UAVs can safely make turns at intersections.

### 4.1. Multi-Layered Air Corridor Design

**Figure 2:**
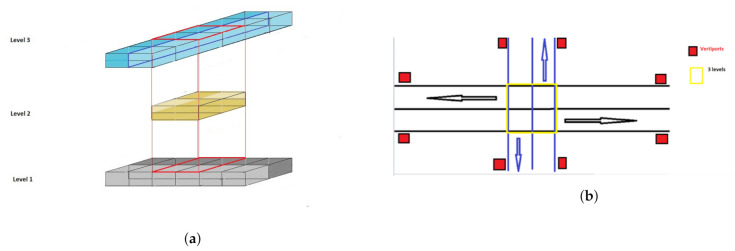
Design of a Multi-layered Air Corridor. (**a**) Side view of an intersection. (**b**) Top view of an intersection: skylanes in level 1 (East-to-West) are in black color, skylanes in level 3 (North-to-South) are in blue color, and red boxes represent vertiports.

In the multi-layered air corridor model, the airspace is divided into two layers throughout the airspace except for intersections (Figure 2). The top layer accommodates southbound and northbound traffic whereas the bottom layer accommodates eastbound and westbound traffic. At intersections, there is also a middle layer which is used by the vehicles for hovering when changing directions. Each layer is represented by its floor and ceiling altitudes $\left\{Z_{f}\left[k\right],Z_{c}\left[k\right]\right\}$, k = 1, 2, 3.

### 4.2. Basic Rules of Engagement

The directions of the skylanes are fixed in top and bottom layers. As a result, the vehicles could move only in the predefined directions in these two layers. If a vehicle wants to change its direction, it needs to come to the middle layer, change its direction, and then go to the desired layer. If the air cube at the desired level is occupied, the vehicle has to wait in the middle layer briefly until the air cube becomes available. The main purpose of middle layer is to avoid collision while vehicles make their turns. This design puts the burden of collision avoidance on the vehicles. As long as the following rules of engagement are enforced, collisions can be avoided: (1) At any given time, a cube can be occupied by only one vehicle. (2) A vehicle needs to make sure that the air cube it is entering at time (*t* + 1) is going to be empty at time (*t* + 1). (3) Overtaking does not occur in air corridor. If one UAV slows down, the UAVs at the rear need to slow down.

### 4.3. Flight Path from One Vertiport to Another

**Figure 3:**
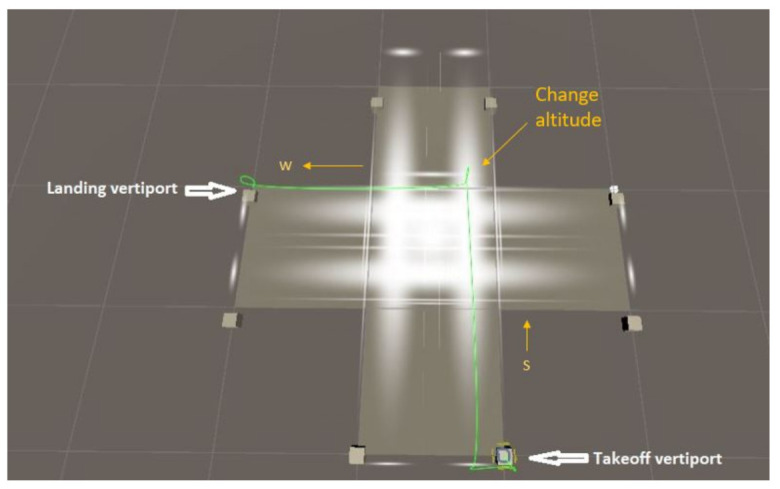
Flight path from south vertiport to west vertiport in multi-layered air corridor.

If a vehicle wants to travel from the south vertiport to the west vertiport, it will use the skylane from south to north at level 3, travel to an intersection, change its altitude to level 2. If level 1 is empty at (next) time instant (*t* + 1), then the vehicle will go to level 1 switching to the westbound skylane to reach its destination. Otherwise, the vehicle will hover in the middle layer until level 1 becomes empty. The route is indicated in green in Figure 3.

## 5. Intersection Handling

Intersections contain three levels to support vehicles while they change the direction of flight. When a vehicle needs to make a turn, it first goes to the middle layer (level 2), changes its heading and then goes to the desired level. Before going to the desired level, the vehicle will check the occupancy of its desired level at the next time step. If the desired level (1 or 3) is occupied by another UAV at that time, the UAV hovers in the middle layer until the desired level is empty.

**Figure 4:**
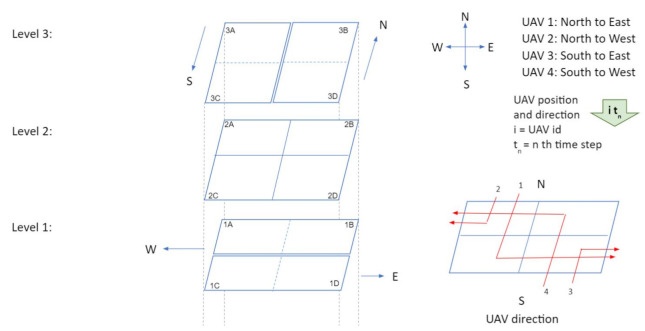
Designing traffic intersections in air corridors.

**Figure 5:**
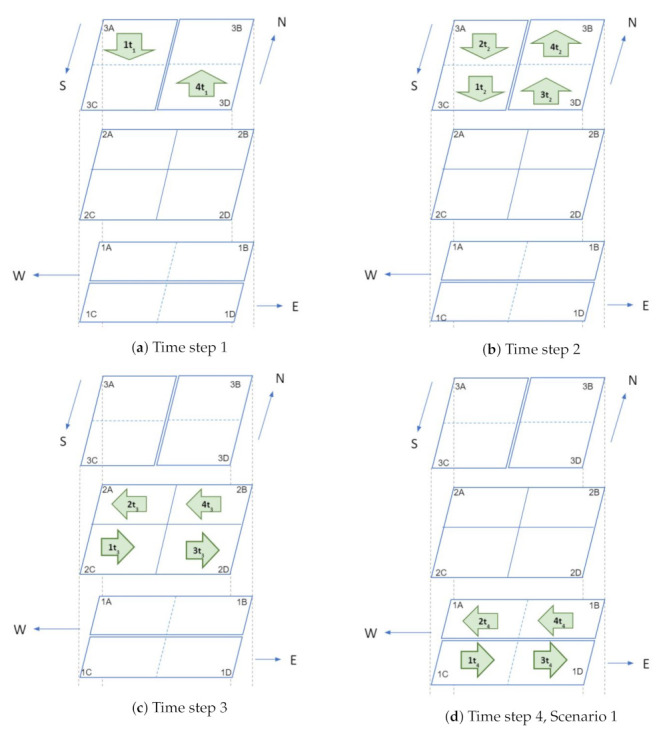
Traffic management at intersections: first illustration.

**Figure 6:**
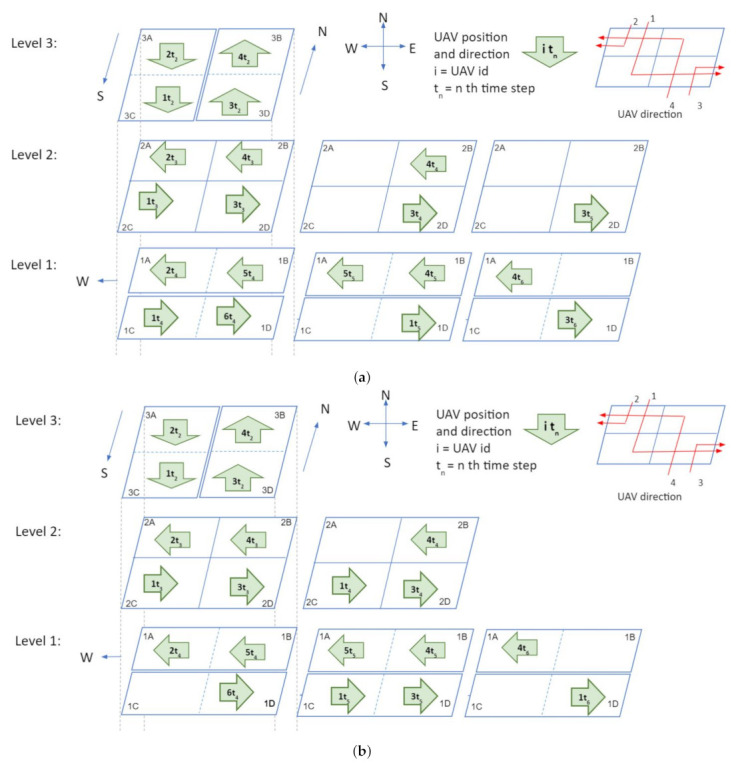
Traffic management at intersections: second illustration. (**a**) Time step 4: scenario 2, approach 1. (**b**) Time step 4: scenario 2, approach 2.

Figure 4 illustrates a three level intersection in which each level contains four air cubes. The four cubes in level 1 are 1A, 1B (for westbound vehicles), 1C and 1D (for eastbound vehicles); cubes in level 3 are labeled as 3A, 3C (for southbound vehicles), 3B and 3D (for northbound vehicles); Cubes in level 2 are 2A, 2B, 2C, and 2D and these cubes are used for hovering. Traffic patterns at intersections are illustrated with four examples in Figure 5 and Figure 6. Here, four UAVs are simultaneously turning at the intersection: (1) UAV 1 is turning from north to east, (2) UAV 2 is turning from north to west, (3) UAV 3 turning from south to east, and (4) UAV 4 is south to west. For simplicity, gap (*g*) between cubes is set to zero. The traffic management at intersection is outlined in the following four steps.

1. Time step 1: At time $t_{1}$, only two vehicles are inside the intersection: UAV 1 in cube 3A and UAV 4 in cube 3D (Figure 5a).
2. Time step 2: In step 2 ($t_{2}$), all vehicles move one cube further. Thus, UAVs 2 and 1 go to cubes 3A and 3C, respectively. Similarly, UAVs 3 and 4 go to cubes 3D and 3B respectively (Figure 5b).
3. Time step 3: In this step, all vehicles will change their heading direction to make a turn. AT time step 3 ($t_{3}$), all four UAVs will change their altitude and move to level 2 from level 3 (Figure 5c).
4. Time step 4: Two possible scenarios arise in step 4. In the first scenario, the UAVs observe that all cubes in level 1 are empty. As a result, the UAVs will move to level 1 at time step 4, while the UAV position remains constant but the altitude changes (Figure 5d). In the second scenario, assume that cubes 1B and 1D in level 1 are occupied by UAV 5 and UAV 6 respectively, at time step 4. So the negotiation between the UAVs can be carried out in two possible ways. In the first approach (Figure 6a), both UAV 3 and 4 will stay in level 2 (in cubes 2D and 2B), and UAV 1 and UAV 2 will move to level 1 (to cubes 1A and 1C) at step 4 . At time step 5; UAV 2, UAV 5, and UAV 1 will move one cube further. Thus UAV 5 and UAV 1 will be at cubes 1A and 1D, respectively, which gives UAV 4 the opportunity to move to cube 1B. UAV 3 will still be hovering in the second level at time step 5. UAV 3 will move towards level 1 at time step 6. In this negotiation process, the UAV that finds an empty cube first, will get the right of way. In the second approach (Figure 6b), UAVs 1, 3, and 4 will stay at level 2 (in cubes 2A, 2D, and 2B) and UAV 2 will move to level 1 (1A) at time step 4. UAV 4, UAV 1, and UAV 3 will go to level 1 at time step 5 and keep moving towards their destination. In this negotiation procedure, UAVs in the front get priority over the ones that are behind. This intersection design allows implementation and utilization of multiple negotiation procedures.

## 6. Capacity of an Air Corridor

In this section, the concept of the capacity of an air corridor is introduced and analyzed. The notion of the capacity of an air corridor captures the idea of how many vehicles can safely fly in a given volume of airspace.

**Definition** **8** (Capacity)**.** *Capacity of an air corridor is defined by the maximum number of vehicles that can fly in the corridor maintaining minimum safe distance among them.*

Consider a unit cube with 1 unit volume and assume that it is divided into smaller cubes of side length *s*. If one UAV is allowed in each smaller cube, the capacity of the unit cube is would be $\frac{1}{s^{3}}$ where s<<1. In the three-layered air corridor model described in Section 4, the top and bottom layers are utilized for traffic and the middle layer is used for hosting the vehicles preparing for turns. In this model, the available space in the air corridor is limited to two layers. Hence the capacity of the air corridor is $\frac{2}{3\times s^{3}}$ where s<<1. If one considers a skylane, the available airspace depends on the length of the skylane (*L*), the side length of the air cube (*s*) and the minimum gap between the UAVs in terms of number of cubes (gc). Thus, the capacity ($C_{skylane}$) of a skylane can be computed as

(5) $$C_{skylane}=\left\{\frac{L}{(gc+1)\times s}\right\}.$$

For example, if the length of a skylane is 10 km, the side length of each cube is 200 m, and the gap between two vehicles is one cube, the capacity of the skylane is going to be 20.

### Travel Time

Assuming that the length of a cube *s* and there are nc number of cubes in a route, the travel time for a UAV to complete the route is given by,

(6) $$Travel\;Time,\;T\;=\;\frac{s\cdot nc}{u}+T_{delay},$$

where *u* represents the vehicle speed, and $T_{delay}$ takes into account delays during takeoff, landing, and waiting time during the travel. In estimating the travel times, it is convenient to consider intervals rather than fixed values for vehicle speeds and delays. For example, the speed of a vehicle *u* ∈ $\left[u_{min},u_{max}\right]$ and the flight delay $T_{delay}$ ∈ $\left[T_{delay-min},T_{delay-max}\right]$.

## 7. Mobility Model and Stationary Node Distribution

The distribution of vehicle locations plays an important role in many computations related to traffic modeling. Estimation of vehicle location distribution requires a mobility model. Among the existing mobility models, the authors found the Manhattan Mobility Model [14] to be the most suitable for modeling traffic in air corridors.

### 7.1. Manhattan Mobility Model with Safety Distance Rules

**Figure 7:**
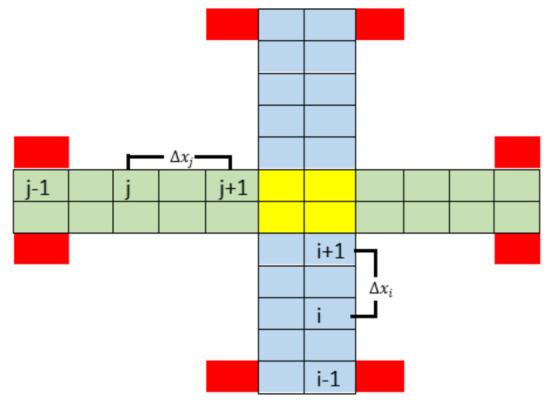
North-to-south skylanes are represented in blue color, east-to-west skylanes are represented in green color, intersections are shown in yellow color, and vertiports are colored in red. Indices i−1, *i*, and i+1 represent three vehicles in level three and the distance between any pair of vehicles is $\Delta x_{i}$. Similarly, *j*, j+1, and j−1 represent three vehicles in level one and the distance between any pair of vehicles in this level is $\Delta x_{j}$.

Figure 7 illustrates the Manhattan mobility model in an air corridor with four skylanes. South-to-north directional vehicles are colored in blue and east-to-west directional vehicles are colored in green. The intersection is shown in yellow color. At a given time instant *t*, vehicle *i* is at position $x_{i}\left(t\right)$, and the speed of vehicle *i* is $u_{i}\left(t\right)$. The vehicles in front and back of vehicle *i* are represented as (*i* + 1) and (*i*− 1) located at $x_{i+1}$ and $x_{i-1}$ with velocities $u_{i+1}$ and $u_{i-1}$, respectively. The distance between *i* and *i* + 1 is $\Delta x_{i}$. Similarly, j represents another vehicle moving from west to east and the vehicles in front and back of *j* are *j* + 1 and *j* − 1 respectively. The distance between *j* and *j* + 1 is $\Delta x_{j}$.

The Manhattan mobility model includes a minimum safety distance (SD) requirement between vehicles, which is implemented in the lanes. Let η be a random variable (RV) uniformly distributed in [−1,1] which adds randomness to the vehicle speed. The speed $u_{i}$ of vehicle *i* is also a uniform RV in the interval $\left[u_{min},u_{max}\right]$ and α denotes the acceleration of vehicles.

(7)ui(t+Δt)=ui(t)+ηαΔt(8)Ifui(t)>umax,thenui(t)=umax;(9)Ifui(t)<umin,thenui(t)=umin;(10)Δxi(t)≤SD,thenui(t)=ui+1(t)−α/2;

Equation (7) represents vehicles speed where η adds some randomness. Equations (8) and (9) limits the vehicles speed inside the corridor in the interval of $\left[u_{min},u_{max}\right]$. Equation (10) provides the safety distance rules. If two UAVs become closer than the minimal SD, the UAV at the rear slows down.

### 7.2. Probability Density Function of UAV Locations

The probability density function of the location (X[t]) of UAVs, is given by,

(11) $$\lim_{t\to \infty }f\left(X\left[t\right]=c\left[cid\right],1\leq cid\leq nc\right)=\frac{1}{nc},$$

where the length of each skylane represented in terms of the number of air cubes is nc. Expressed in terms of distance, the length (*L*) of a skylane is the product of number of cells (nc) and the side length of a cube (s), i.e., L=nc·s. From (11), it can be deduced that the probability of an air cube being occupied by a UAV is given by $\frac{N}{nc}$, N≤nc, where *N* is the total number of UAVs. Lemma 1 suggests that the node distribution of UAV locations remains uniform as long as there are no obstacles in the skylane.

**Lemma** **1.** *The Manhattan grid model leads to a uniform distribution of locations of vehicles in a skylane. Traffic may be slow or fast, but it always flows as long as there are no obstacles in the skylane.*

The proof follows the Manhattan mobility Model with SD requirement described in (7) and the uniform distribution of both u(t) and η.

## 8. Collision Probability

**Definition** **9** (Collision Probability)**.** *Collisions occur when two or more UAVs occupy a cube at the same time.*

Collision probability is the probability of one cube occupied by more than one UAV at the same time. One way to avoid any potential collision is to enforce the rule that there can be only one vehicle in a cube. This can be accomplished with the safety distance rule described in the mobility model (7). However, collision can occur for many unforeseen situations including congestion at intersections or vehicle failures. Lemma 2 estimates the probability of no collision as a function of number of UAVs present in a skylane.

**Lemma** **2.** *Assume that there are N number of UAVs and nc number of cubes in a skylane and N<<nc. Then, the probability of no collision ($P_{no-collision}$) is given by:*

(12) $$P_{no-collision}=\frac{\left(nc)*(nc-1)*(nc-2)*\ldots *(nc-(N-1)\right)}{{\left(nc\right)}^{N}}\;∼\;\frac{{\left(nc\right)}^{nc-N+1/2}}{e^{N}\cdot {\left(nc-N\right)}^{nc-N+1/2}}$$

**Proof.**  With the restriction that only one UAV can be present in one cube at any given time, the number of ways *N* UAVs can be in nc number of cubes is given by:

(13) $${}^{nc}P{}_{N}=\frac{nc!}{(nc-N)!}.$$

This expression forms the numerator for the first expression on the right hand side of (12). If any UAV is allowed to occupy any cube without restriction, then the number of ways *N* vehicles occupy nc cubes is given by ${\left(nc\right)}^{N}$. This forms the denominator for the first expression on the right hand side of (12). If both *N* and (N−nc) are large numbers, we can use Sterling’s formula ($n!∼\;{\sqrt{2\pi n}\;\left(\frac{n}{e}\right)}^{n}$) to approximate the factorials. With this approximation, we can rewrite (14) as follows:

(14) $${}^{nc}P{}_{N}=\frac{nc!}{(nc-N)!}\;∼\;\frac{{\left(nc\right)}^{nc+\frac{1}{2}}}{e^{N}\cdot {\left(nc-N\right)}^{nc-N+\frac{1}{2}}}$$

(15) $$\begin{array}{cc} Probability\;of\;no\;collision & \;=\frac{Number\;of\;options\;with\;restriction}{Number\;of\;options\;without\;restriction}\\ & \;=\frac{{}^{nc}P{}_{N}}{{\left(nc\right)}^{N}}\\ & \;=\frac{\frac{nc!}{(nc-N)!}}{{\left(nc\right)}^{N}}\\ & \;∼\;\frac{{\left(nc\right)}^{nc-N+\frac{1}{2}}}{e^{N}\cdot {\left(nc-N\right)}^{nc-N+\frac{1}{2}}} \end{array}$$

□

**Figure 8:**
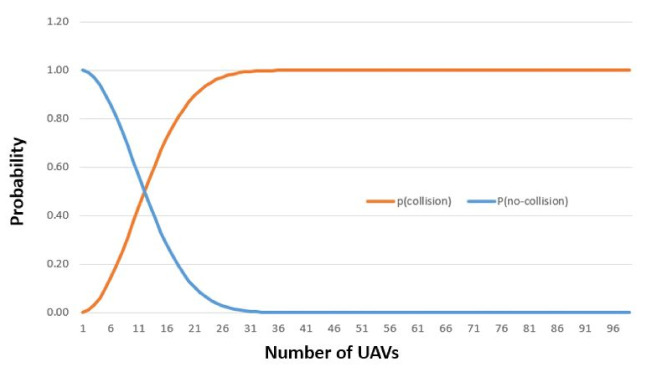
$P_{collision}$ and $P_{no-collision}$ vs. Number of UAVs when nc=100.

Figure 8 illustrates the collision probability with nc set to 100. It also suggests that the best way of avoiding collisions is by enforcing the rule that there can only be one UAV in one cube.

## 9. Simulations and Results

This section discusses the results of discrete-time simulations carried out to demonstrate the long-term distribution of UAV locations within a skylane as a function of system parameters such as the velocity of the UAVs, traffic volume, and length of time steps. For simplicity of the simulation, the skylane is considered as a one-dimensional grid. Vehicles can move from one cube to another at a time in one direction. Since vehicles have varying speeds, some vehicles may reach the next cube between time steps and some vehicles may not. Vehicles moving at the maximum speed may advance at most one air cube per time step.

**Figure 9:**
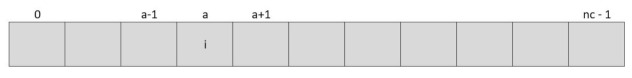
Traffic flow in a skylane with nc cubes.

Figure 9 depicts the skylane with nc air cubes. Traffic flows from the left to the right, starting from cube 0. A new vehicle can enter cube 0 only when it is not occupied. Every vehicle leaves the skylane from cube nc−1 and presence of obstacles is not taken into account. The Manhattan mobility model allows vehicles to move forward with a given probability ($P_{move}$) if the next cube is vacant. For a vehicle *i* in cube *a* at time *t*, the probabilities for the next time step t+1 are:

(16) $$P_{i,t+1}\left(x=a+1\right)\;=\left\{\begin{array}{cc} 0, & \mathrm{if}\;a\;+\;1\;\mathrm{occupied}\\ P_{move}, & \mathrm{otherwise} \end{array}\right.$$

### 9.1. Simulation Comparing Different Velocities

Instead of simulating variations in vehicle’s velocity, the transition probabilities ($P_{stay}$ and $P_{move}$) for a vehicle to advance to the next cube are defined. Higher probability of vehicle staying in the same cube ($P_{stay}$) indicates that the traffic moves slowly. For fast moving vehicles, $P_{stay}$in [0.001, 0.050] and for slow moving vehicles $P_{stay}$in [0.20, 0.90]. The probability of a vehicle moving to the next cube, $P_{move}\;=\;1-P_{stay}$. The simulation was run for 100,000 time steps considering 20 vehicles in a skylane consisting of 100 air cubes.

#### 9.1.1. Convergence

**Figure 10:**
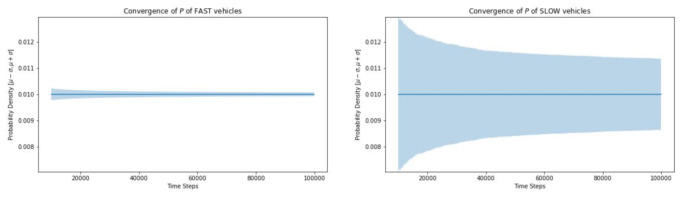
Convergence of fast vs. slow moving vehicles.

The standard deviation for the probability density function is much greater for slow traffic and the simulation takes much longer to converge (Figure 10).

#### 9.1.2. Vehicle Distribution along the Skylane

**Figure 11:**
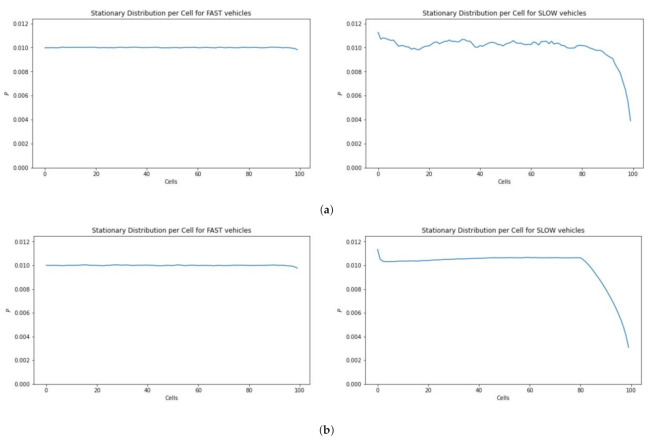
Location distribution of fast vs. slow moving vehicles. (**a**) Time steps = 100,000. (**b**) Time steps = 10,000,000.

Figure 11 depicts the occupancy information of 100 cubes having 20 UAVs. The simulation results show that in the case of slow traffic the cubes at the end of the skylane are less likely to be occupied (Figure 11a) even with more simulation steps (Figure 11b).

#### 9.1.3. Trajectories

**Figure 12:**
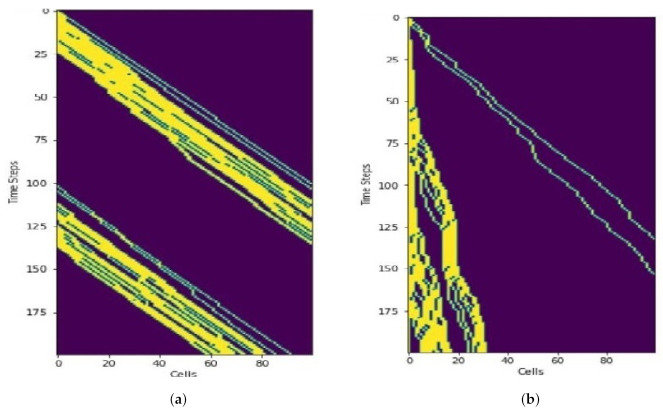
Trajectories of fast vs. slow moving vehicles. Only the first 200 time steps are shown. (**a**) Fast moving vehicles. (**b**) Slow moving vehicles.

Simulation results (Figure 12) show how the vehicles move along the corridor in horizontal axis. The vertical axis depicts the time. Unlike road traffic, overtaking does not occur in air corridor. As a result, even a single slow vehicle impacts the traffic flow. Cubes at the end of the skylane become occupied quckly in the case of fast moving UAVs (Figure 12a). On the other hand, Figure 12b depicts that the end of the skylane needs more time to become occupied if the UAVs are moving slowly.

### 9.2. Simulation Comparing Different Traffic Volumes

Traffic volume, i.e., the ratio of vehicles to air cubes in skylane ($\frac{N}{nc}$) is analyzed here. Simulation results are shown for 10 and 50 UAVs in 100 air cubes, which correspond to the ratios of vehicles to cubes 0.1 and 0.5, respectively.

#### 9.2.1. Probability Density

**Figure 13:**
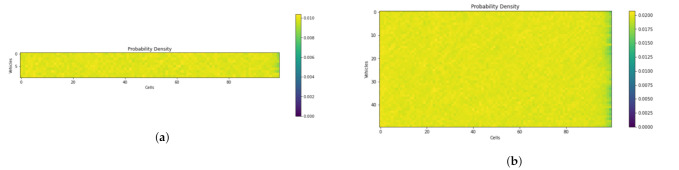
Probability density for different traffic volumes. (**a**) Ratio of vehicles to air cubes in skylane ($\frac{N}{nc}$) = 0.1. (**b**) Ratio of vehicles to air cubes in skylane ($\frac{N}{nc}$) = 0.5.

The horizontal axis in Figure 13 depicts the number of cubes, while the vertical axis represents the number of UAVs. The number of UAVs is represented by the color density. The simulation results show that the probability density along the corridor is uniform despite the variances in traffic volume.

#### 9.2.2. Convergence

**Figure 14:**
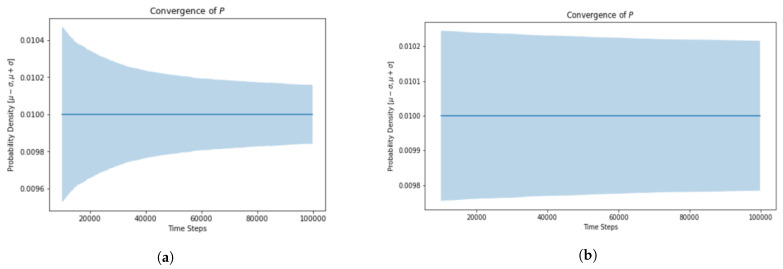
Convergence for different traffic volumes. (**a**) Ratio of vehicles to air cubes in skylane ($\frac{N}{nc}$) = 0.1. (**b**) Ratio of vehicles to air cubes in skylane ($\frac{N}{nc}$) = 0.5.

For low traffic volume, the standard deviation of the probability density function is substantially higher, and the simulation takes much longer to converge. Higher traffic volume, on the other hand, requires less time to converge. (Figure 14).

#### 9.2.3. Location Distribution along the Skylane

**Figure 15:**
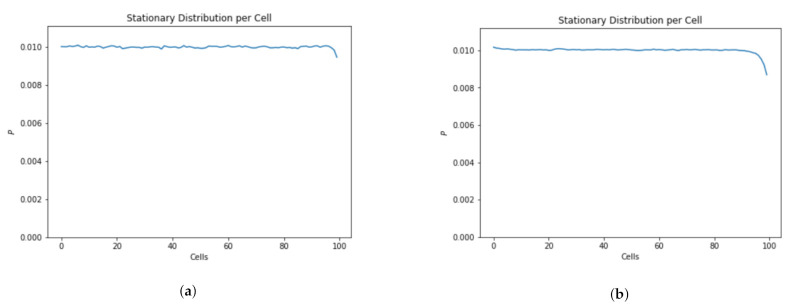
Location distribution along the skylane for different traffic volumes. (**a**) Ratio of vehicles to air cubes in skylane ($\frac{N}{nc}$) = 0.1. (**b**) Ratio of vehicles to air cubes in skylane ($\frac{N}{nc}$) = 0.5.

Figure 15 shows that vehicle location distribution along the skylane is going to be stationary for different traffic volumes.

#### 9.2.4. Trajectory

**Figure 16:**
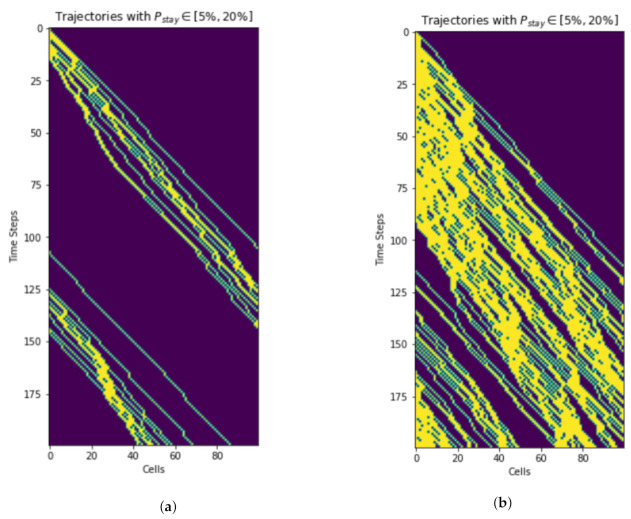
Vehicle trajectory analysis for different traffic volumes. Only the first 200 time steps are shown. (**a**) Ratio of vehicles to air cubes in skylane ($\frac{N}{nc}$) = 0.1. (**b**) Ratio of vehicles to air cubes in skylane ($\frac{N}{nc}$) = 0.5.

Higher traffic levels mean more UAVs, therefore the trajectory is more crowded than at lower traffic volumes. (Figure 16).

## 10. Summary and Conclusions

This paper provided formal definitions for air corridor and its constituent building blocks including air cubes, skylanes, intersections, vertiports, and gates. Rules of engagement for the collision-free traffic inside the corridors are discussed. Traffic management at intersections is illustrated with a few examples. The notion of capacity of skylane as a function of number of cubes and gap size between UAVs is presented. Probability of collision and probability density of locations of vehicles in air corridors are discussed. Simulations of traffic in air corridors are presented to demonstrate that the distribution of vehicle locations remains uniform despite variations in traffic volumes or vehicle speeds.

## Figures and Tables

**Table 1:** Notation.

Symbol	Description	Symbol	Description
S[]	List of skylanes	sid	Skylane identifier
ids[ ]	List of identification numbers of the permitted UAVs	gc	Gap between UAVs in terms of number of air cubes
V[ ]	Vertiport	vpid	Vertiport identifier
c[ ]	List of air cubes	cid	Air cube identifier
nc	Number of air cube	s	Side length of air cube
g	Geofence	$h_{i}$	Home location
n	Number of vertices in geofence	*v*	List of vertices in geofence
$z_{f}$	Minimum floor altitude	$z_{c}$	Maximum ceiling altitude
ϕ	Latitude	λ	Longitude
$z_{i}$	Altitude	$t_{i}$	Activation time
C	Capacity	N	Number of UAVs
l	Number of skylanes	L	Length of a skylane
T	Travel time	$T_{delay}$	Delay time
SD	Safety distance	α	Acceleration
$u_{max}$	Maximum speed	$u_{min}$	Minimum speed
$u_{i}$	Speed of vehicle i	$x_{i}$	Position of vehicle i

## Data Availability

https://github.com/SabrinaIslamMuna/AirCorridor.git (accessed on 11 November 2021).

## Abbreviations

The following abbreviations are used in this manuscript:

UAV	Unmanned Air Vehicle
UAS	Unmanned Aircraft System
AAM	Advanced Air Mobility
UAM	Urban Air Mobility
UTM	UAS Traffic Management
FAA	Federal Aviation Administration
BS	Base Station
NFZ	No-Fly Zone
MSL	Mean Sea Level
MAC	Mid-Air-Collision
